# Pharmacy Education; Competency and Beyond

**DOI:** 10.3390/pharmacy8020104

**Published:** 2020-06-19

**Authors:** Martin C. Henman

**Affiliations:** School of Pharmacy and Pharmaceutical Sciences, Trinity College Dublin, Dublin D02, Ireland; MHENMAN@tcd.ie

Competency-based curricula are the dominant approach in the education of healthcare professionals around the world today.

Competency assessment is a behaviourist approach. It necessitates the defining of all the behaviours required in demonstrating competency. This can be a valuable exercise in exploring, in detail, what each competency entails. When this is applied to an entire course, it is necessary to map and reconcile the competencies that are required at the module, course and programme levels. Once again, this is valuable because it ensures a comprehensive and structured view of the programme and all of its parts.

Nevertheless, identifying the behaviours for each competency is only one part of the process. The learning outcomes and the competencies must be aligned, and they must be underpinned by a teaching and assessment strategy that provides the staff and students with a clear set of resources and procedures that enable the learning outcomes to be attained and the competencies to be demonstrated. Finally, the systematic application of rigorous methods to quality-assure these outcomes validates the processes.

Self-evidently, this brief description shows the complexity and the scale of the work that must be done to deliver a competency-based curriculum. It is no wonder that academics have seen an increase in administration and management activities.

However, several other points also emerge:

First of all, the competency-based approach needs to be enacted in all parts of the curriculum. It is not enough to evaluate behaviours in the clinical subjects and to ignore them elsewhere—if different standards are applied in one subject compared to another, then some people will behave differently. Behaviours, and the standards that are set, are part of the ‘culture’, and without consistency throughout healthcare, poor practices can become entrenched and accepted.

Secondly, aligning learning outcomes with competencies helps to clarify the objectives of each element in the course, and mapping these across the entire course and programme helps to establish the appropriate level of attainment at each stage, and to structure the sequencing of the delivery of subject matter. This is critical because, as biomedical knowledge expands, and as the scope of practice and the responsibility of pharmacists grow, greater co-ordination and integration within the curriculum is essential [1].

Thirdly, teaching and assessment strategies are fundamental. They are more complicated in healthcare programmes than in many other university courses, because they must take account of periods of experiential learning in practice and other placements. Not only must suitable assessments be devised for developing student’s critical appraisal skills, they must also be prepared for placements using simulations; assessed during them, using workplace techniques; and finally, they need to show that they have integrated these learnings via the use of reflection [2].

Fourthly, accreditation involves the assuring of the structures, procedures, outcomes and organisational culture of the programme, and this too has grown in scale and complexity in response to the changes in pharmacy education. 

Competency and its assessment is not, by itself, sufficient to define a pharmacy programme or a pharmacist. Each country has its own approach to competency-based curricula and to the accreditation of those curricula, and this has led to a range of strategies and policies. The scope of practice of pharmacists is developing in different ways and at different speeds around the world [3]. This is exacerbated by the lack of agreement among pharmacists as to their role, and by the roles assigned to pharmacists in different healthcare systems. While each country can pursue its own course, there are many ideas and ideals that are held in common. It is therefore possible and important to seek a consensus, to enable the development of a profession that contributes so much to patient care and to the development of new medicines and medical technology [4]. Within pharmacy, through organisations such as FIP (International Pharmaceutical Federation), consensus documents [5] and a framework of quality assurance [6] have ensured that discussion and debate have taken place, ideas have been exchanged and resources created to facilitate the evolution of pharmacy education. However, pharmacy and pharmacists must collaborate with patients and with healthcare professionals so that humanistic values [7] and interprofessional education [8] do not remain underdeveloped.

This Special Issue demonstrates the multiplicity of curricula developments being undertaken and the innovations that are being implemented in pharmacy education today. They illustrate that the work to establish satisfactory competency-based curricula is, inevitably, continuing alongside the other activities necessary to assure that pharmacy programmes meet the needs of the communities that they serve, and that pharmacy educators are looking beyond competency to attain this objective. 

This can lead to long lists of behaviours for one competency, each of which must be demonstrated in order for a student to be regarded as competent. These activities are valuable, indeed, essential components of health sciences education.
